# Molecular epidemiological and clinical infection characteristics analysis of *Ralstonia*

**DOI:** 10.1007/s10096-024-04823-w

**Published:** 2024-04-19

**Authors:** Zhaojun Sheng, Jiaxin Li, Guojing Han, Ru Fan, Pingjun Zhu, Xiangqun Fang

**Affiliations:** 1 College of Pulmonary and Critical Care Medicine, Chinese PLA General Hospital, Beijing, China; 2 Department of Respiratory and Critical Care Medicine, Aerospace Center Hospital, Beijing, China; 3 Department of Respiratory and Critical Care Medicine, The Second Medical Center & National Clinical Research Center for Geriatric Diseases, Chinese PLA General Hospital, Beijing, China

**Keywords:** *Ralstonia pickettii*, Bacterial identification, MLST, Antibiotic susceptibility, Clinical symptoms

## Abstract

**Purpose:**

This study was to clarify the molecular epidemiology and clinical infection characteristics of *Ralstonia pickettii* and establish sequence typing system.

**Methods:**

48 nonrepetitive *Ralstonia pickettii* strains were collected from January 2008 to December 2013 at the Chinese People’s Liberation Army General Hospital (PLAGH) and were identified through a specific PCR experiment, 16 S rDNA experiment and VITEK 2 system to compare the identification accuracy. The sequence types of the strains were analyzed by multilocus sequence typing (MLST) method. The antibiotic sensitivity of these strains was determined with disc diffusion tests and broth microdilution method. The clinical data of *Ralstonia pickettii* infected patients were collected.

**Results:**

All of the 48 strains were identified as *Ralstonia pickettii* by VITEK 2 system. 30 and 34 strains were identified as *Ralstonia pickettii* by PCR and 16 S rDNA experiment respectively. ST9 was the most sequence types (STs) in these 18 STs of 42 strains. 42 strains were divided into 2 groups (A and B) and 18 genotypes. *Ralstonia pickettii* was sensitive to some cephalosporins, β-lactam/β-lactamase inhibitor, levofloxacin and trimethoprim/sulfamethoxazole. Cough, sputum, shortness of breath and pulmonary rales were the common clinical symptoms of most *Ralstonia pickettii* infected patients.

**Conclusion:**

We established a sequence typing system with a relatively fine resolution and the PCR assay is a faster and more sensitive method for clinical identification of *Ralstonia pickettii*. ST9 is the most common sequence types of *Ralstonia pickettii*. The most common clinical characteristics of *Ralstonia pickettii* infected patients were cough, sputum, shortness of breath and pulmonary rales.

## Introduction

*Ralstonia pickettii*, as a Gram-negative aerobic bacillus, was first isolated and ascribed to *Burkholderia cepacian* in 1973. Due to its phenotypic characteristics, cellular lipid and fatty acid analysis, rRNA–DNA hybridization and phylogenetic analysis of 16s rDNA nucleotide sequences, the genus was separated from *Burkholderia spp* and was ascribed to *Ralstonia Spp* in 1995 [1]. The most common clinically pathogenic pathogens in that species are *Ralstonia pickettii* (*R. pickettii*), *Ralstonia mannitolilytica* (*R. mannitolilytica*) and *Ralstonia insidiosa (R. insidiosa*). *Ralstonia pickettii* has been isolated from a wide variety of clinical specimens including blood, urine and cerebrospinal fluid [2]. The organism may be a more widespread pathogen and the types of infections are more invasive and severe than previously thought [3–6].

The existing commercial biochemical identification systems on the market, e.g. API ^@^20 NE test system (BioMe ´rieux, Marcy l’Etoile, France) and Rap ID NE, do not always give the most dependable identification. *Ralstonia pickettii* has been shown to give variable results using the standard biochemical test kits, e.g. API 20NE test system [7]. Due to the similarity between *Ralstonia pickettii* and the *B. cepacia* complex, it is also thought that many cases of the *B. cepacia* complex may have been misidentified and are in fact *Ralstonia pickettii*. *Ralstonia pickettii* has been also associated with nosocomial outbreaks caused by contaminated solutions used for patient care and with pseudo epidemics caused by contaminated solutions in the diagnostic laboratory [8–12]. In addition, the treatment of *Ralstonia pickettii* infections is often challenging as this bacterium has been reported as being intrinsically resistant to many antimicrobial agents [13], which could be due to the presence of mobile genetic elements [14]. There is limited information on the surveillance and monitoring of the antibiotic resistance of these bacteria.

In this study we initially established a program for MLST to facilitate origin-tracing of infections and study of population structures and explored a sensitive way to identify *Ralstonia pickettii*. Meanwhile, we reported the antimicrobial susceptibility of *Ralstonia pickettii* isolates and analysed the clinical symptoms of *Ralstonia pickettii* infected patients, to provide reference for the treatment of clinical infection patients.

## Materials and methods

### Strains

48 non-repetitive *Ralstonia pickettii* strains were isolated from patients hospitalized from January 2008 to December 2013, which were identified by VITEK 2 system (BioMérieux, Marcy l’Étoile, France).

### Bacteria identification

#### Commercial identification system

Strains were re-identifid according to the instructions of VITEK 2 system (BioMérieux, Marcy l’Étoile, France).

#### PCR experiment

Specific primer 1 for *Ralstonia pickettii* was developed in a previous study [15], while primer 2 was designed for *R. mannitolilytica* and primer 3 was designed for *R. insidiosa* (Table 1) [16]. Amplification was conducted in a programmable thermal cycler (Idaho Technologies Inc., Salt Lake City, Utah). After an initial 5-min denaturation at 95 °C, 30 amplification cycles were completed, each consisting of 40 s at 94 °C, 40 s at 55/57°C (the annealing temperatures used were 55 °C for the identification of *R. insidiosa* and *Ralstonia pickettii* while 57 °C for the identification of *R. mannitolilytica*), and 1 min at 72 °C, followed by an additional final extension of 5 min at 72 °C. Negative PCR controls, containing all reaction mixture components except template DNA, were included for every experiment.

**Table 1:** Primers for the identification of *Ralstonia Spp*. by PCR

Identification bacteria	Primer name	Primer sequence (5’-3’)	Annealing temperature (℃)	Product size (bp)
Primer 1	Rin/Rpi-F1	ATGATCTAGCTTGCTAGATTGAT	55	210
(*Ralstonia pickettii*)	Rpi-R1	ACTGATCGTCGCCTTGGTG		
Primer 2	Rma-F1	GGGAAAGCTTGCTTTCCTGCC	57	398
(*R. mannitolilytica*)	Rma-R1	TCCGGGTATTAACCAGAGCCAT		
Primer 3	Rin/Rpi-F1	ATGATCTAGCTTGCTAGATTGAT	55	403
(R. insidiosa)	Rin-R1	CACACCTAATATTAGTAAGTGCG		

#### 16 S rDNA

The consensus primers were used to perform 16 S rDNA experiment (Table 2) [17]. The amplification was carried out in a thermocycler (Idaho Technologies Inc., Salt Lake City, Utah). After an initial 5-min denaturation at 95 °C, 30 amplification cycles were completed, each consisting of 40 s at 94 °C, 40 s at 50 °C, and 1 min at 72 °C, followed by an additional final extension of 5 min at 72 °C. Perform bi-directional sequencing on amplification products and splice the determined sequence through EditSeq (DNA Star Inc.). Basic local alignment search tool (BLAST) on the spliced sequence was performed in NCBI database (www.ncbi.nlm.nih.gov/BLAST).

**Table 2:** Consensus Primer of 16 S rDNA Experiment

Primer name	Primer sequence (5’-3’)
27f-YM	AGAGTTTGATYMTGGCTCAG
1492r	TACCTTGTTACGACTT

*Notes* Y = T or C, M = A or C, 1500 bp of amplified fragment.

### MLST sequence typing

6 relatively conserved gene locus were chosen to design and synthesize 6 pairs of amplification primers which are presented in Table 3. The amplification was carried out in a thermocycler (Idaho Technologies Inc., Salt Lake City, Utah). After an initial 5-min denaturation at 95 °C, 30 amplification cycles were completed, each consisting of 40 s at 94 °C, 40 s at 56 °C, and 1 min at 72 °C, followed by an additional final extension of 5 min at 72 °C. For each of the selected genes, the sequences from all 42 isolates were compared and allele numbers were assigned to each unique sequence. The alleles present at the six loci define the allelic profile or sequence type (ST). Cluster analysis was performed using the unweighted pair group method with arithmetic mean (UPGMA) method to construct phylogenetic tree.

**Table 3:** Amplification primers of MLST gene locus

Gene name	Primer sequence (5’-3’)	Annealing temperature (℃)
atpD	F: GCCGTGACATCCTGTTCTTC R: CGTCCACCAGCATCTTGAAG	56
gltB	F: TGATGTCGCTCGTCTCGTTC R: GCCTTGGTGAAGTTGTAGATGG	56
gyrB	F: CGAGATCCAGGTCACCATCC R: AGCACGGTCTTGTTCTTGTTG	56
lepA	F: GGAAGCCAATCAGTACGAAGC R: GTCGCCGTTGATAAGGATGTC	56
phaC	F: ATGGGCGGTATCGGAACGG R: AGGCAATCTCGGTCGGCTC	56
recA	F: TAAGAAGGCAGCCACGATGAG R: CGGATCTGGTTGATGAAGATGAC	56

### Antibiotic susceptibility test

Disc diffusion tests and broth microdilution method were used to determine the antibiotic sensitivity of 34 *Ralstonia pickettii* strains identified by 16 S rDNA experiment. All tests were carried out according to Clinical and Laboratory Standards Institute (CLSI) standards (CLSI, 2015) [18]. As there were no CLSI susceptibility breakpoints available for *Ralstonia pickettii*, *R. insidiosa* or *R. mannitolilytica*, the antibiotic susceptibility results were interpreted using the CLSI criteria for *Pseudomonas sp*, *BurkhoIderia cepacian* and *Acinetobacter spp* (CLSI,2015). All results were found to be within recommended limits, demonstrating the validity Of the testing procedures used.

### Clinical features and outcome of the infected patients

We performed a retrospective study on patients infected with *Ralstonia pickettii*. The patients’ data were collected by reviewing electronic medical records. We analyzed the clinical features, treatment and clinical outcome of the patients.

## Results

### The results of bacterial identification

**Fig. 1:**
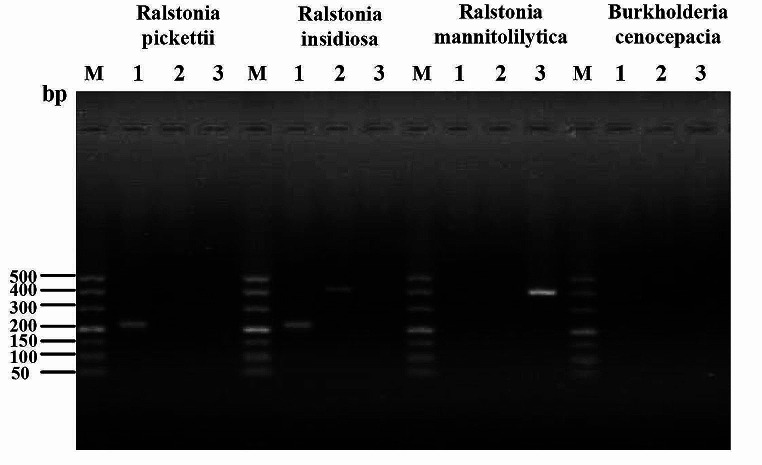
The results of bacterial specific PCR assay. Notes: 1- Primer 1; 2-Primer 2; 3-Primer 3

All 48 strains tested were identified as *Ralstonia pickettii* by VITEK 2 system. There was a band of approximately 210 bp after the amplification of *Ralstonia pickettii* using primer (1) A band of 398 bp around was obtained after the amplification of *R. mannitolilytica* using primer 3, and two bands of about 210 bp and 403 bp were obtained after the amplification of *R. insidiosa* using primer 1 and (2) There was no visible band after the amplification of *Burkholderia cepacian* (See Fig. 1). 30 strains of *Ralstonia pickettii*, 1 strain of *R. insidiosa*, 8 strains of *R. mannitolilytica* were identified by the specific PCR, 9 strains had no target band. After 16 S rDNA identification, there were 34 strains of *Ralstonia pickettii*, 1 strain of *R. insidiosa*, 7 strains of *R. mannitolilytica*, 1 strain of *Achromobacter xylosoxidans*, 2 strains of *Burkholderia cepacia* and 3 strains of *stenotrophomonas maltophilia*.

### Evaluation of VITEK 2 system and PCR assays compared with the 16 S rDNA

Compared with the 16 S rDNA method, the sensitivity of VITEK 2 system was 100%, but the specificity was only 70.8%. As for the specific PCR method, the sensitivity and specificity were 85.3% and 85.7% for *Ralstonia pickettii* respectively,71.4% and 90.2% for *R. mannitolilytica*, both 100% for *R. insidiosa* (Table 4).

**Table 4:** Sensitivity and specificity of PCR assays for the identification of *Ralstonia spp*

Primers	Identification bacteria	Sensitivity (%)	Specificity (%)	Positive strains	Negative strains
Primer 1	*Ralstonia pickettii*(*n* = 34) Others(*n* = 14)	85.3	85.7	29 2	5^a^ 12
Primer 2	*R. mannitolilytica*(*n* = 7) Others(*n* = 41)	71.4	90.2	5 4^c^	2^b^ 37
Primer 3	*R. insidiosa*(*n* = 1) Others(*n* = 47)	100	100	1 0	0 47

*Note*

^a^ Of the 5 Ralstonia pickettii isolates that gave a false-negative reaction in this PCR test, 3 were identified as *R. mannitolilytica*, *1 had no positive target band, 1* cross-reacted with primers 2.

^b^ Of the 2 R. mannitolilytica isolates that gave a false-negative reaction in this PCR test, 1 was identified as Ralstonia pickettii, *1* cross-reacted with primers 1.

^c^ Of the 4 Ralstonia pickettii isolates that gave a false-positive reaction in this PCR test, 3 was identified as R. mannitolilytica, *1* cross-reacted with primers 2.

### MLST sequence typing results

**Fig. 2:**
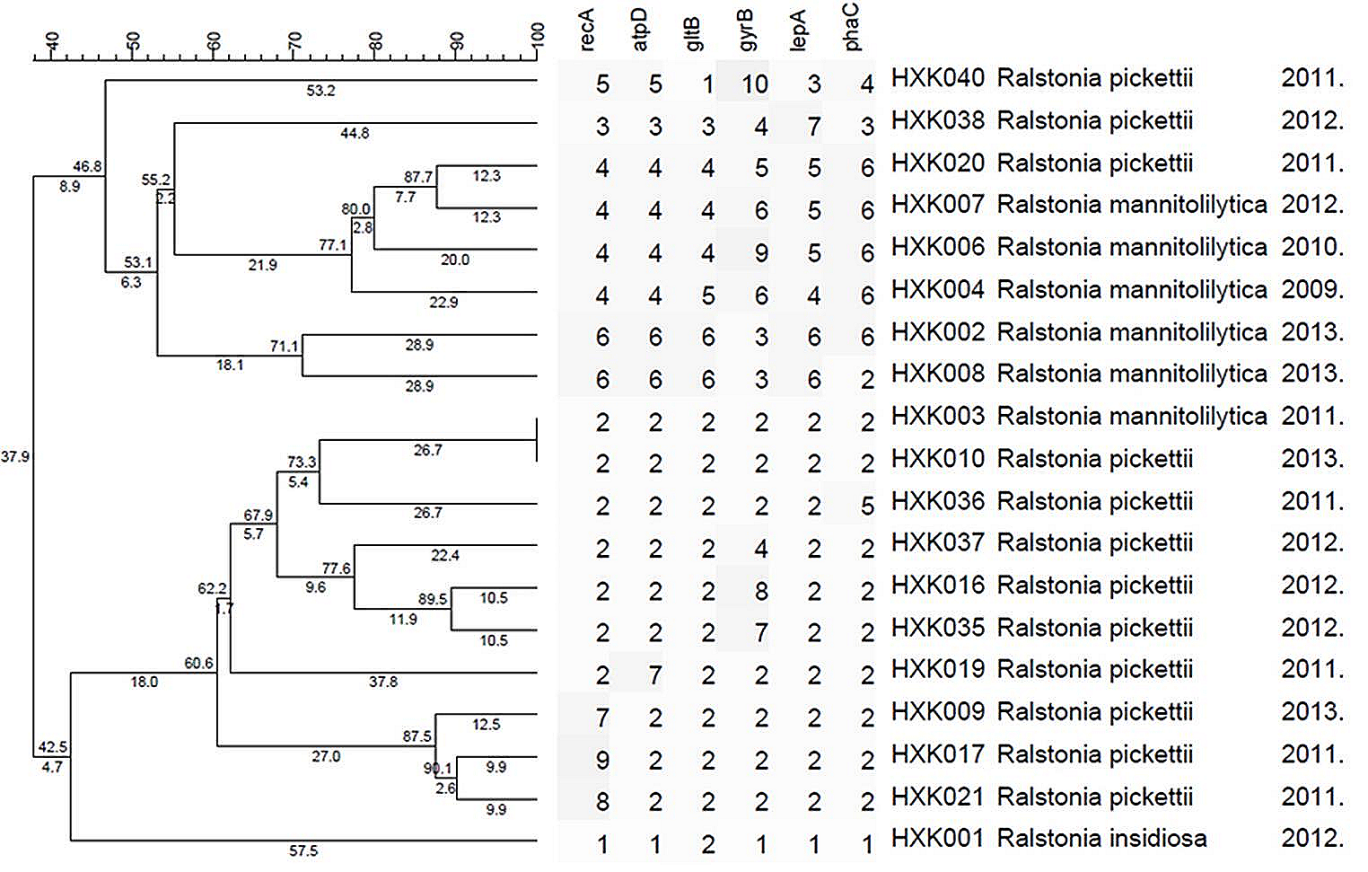
The result of the cluster analysis on 42 strains of *Ralstonia* by UPGMA method

42 strains of *Ralstonia Spp* were analyzed and designated by MLST, resulting in 18 ST types ranging from ST1 to ST18. The most frequent ST type was ST9 with 22 strains which were considered as the advantage type. There are 2 strains were identified as ST2 and ST4, respectively. The result of genotyping by MLST of the 42 strains of *Ralstonia* is presented in Table 5. The result of typing by MLST is presented in Fig. 2. The 42 strains of *Ralstonia* were grouped into 2 groups (A and B) containing 18 genotypes with 8 genotypes in the group A and 10 in the group B respectively. The 8 genotypes in *Group A* contained 10 strains of *Ralstonia*, including 4 strains of *Ralstonia pickettii* and 6 strains of *mannitolilytica*. The 10 genotypes in group B contained 32 strains of *Ralstonia*, including 1 strain of *R. mannitolilytica*,1 train of *R. insidiosa*, and 30strains of *Ralstonia pickettii*.

**Table 5:** The result of genotyping by MLST of 42 strains of *Ralstonia*

Representative strains	Identification result of 16 S rDNA experiment	ST type	Strain numbers
HXK001	*Ralstoniainsidiosa*	ST18	1
HXK002	*Ralstoniamannitolilytica*	ST7	1
HXK003	*Ralstoniamannitolilytica*	ST9	22
HXK004	*Ralstoniamannitolilytica*	ST6	1
HXK006	*Ralstoniamannitolilytica*	ST5	1
HXK007	*Ralstoniamannitolilytica*	ST4	2
HXK008	*Ralstoniamannitolilytica*	ST8	1
HXK009	*Ralstoniapickettii*	ST17	1
HXK016	*Ralstoniapickettii*	ST11	1
HXK017	*Ralstoniapickettii*	ST15	1
HXK019	*Ralstoniapickettii*	ST14	1
HXK020	*Ralstoniapickettii*	ST3	1
HXK021	*Ralstoniapickettii*	ST16	1
HXK035	*Ralstoniapickettii*	ST12	1
HXK036	*Ralstoniapickettii*	ST10	1
HXK037	*Ralstoniapickettii*	ST13	1
HXK039	*Ralstoniapickettii*	ST2	2
HXK040	*Ralstoniapickettii*	ST1	1
HXK042	*Ralstoniapickettii*	ST9	22

### The results of antibiotic susceptibility test

#### Antibiotic sensitivity of Ralstonia pickettii by the disc diffusion tests

All of 48 strains were sensitive to Cefoperazone/sulbactam, Cefepime, Ciprofloxacin, Levofloxacin and Trimethoprim/Sulfamethoxazole. Followed by Piperacillin/tazobactam (94.1%), Minocycline (94.1%) and Ceftazidime (82.4%). However, all the strains were resistant to Aztreonam and Meropenem, while most of the strains were found to be less responsive to Cefoperazone (2.9%), Amikacin (14.7%%), Gentamicin (14.7%) and Polymyxin B (14.7%) (Table 6).

**Table 6:** The antibiotic sensitivity of *Ralstonia pickettii* by disc diffusion tests (%)

Antibacterial Drugs	Drug resistant		Intermediary agent		Sensitive	
	Strain numbers	Resistance rate	Strain numbers	Intermediate rate	Strain numbers	Sensitive rate
Piperacillin	8	23.5	1	2.9	25	73.5
Piperacillin/tazobactam	2	5.9	0	0.0	32	94.1
Aztreonam	33	97.1	1	2.9	0	0.0
Cefoperazone	31	91.2	2	5.9	1	2.9
Cefoperazone/sulbactam	0	0.0	0	0.0	34	100.0
Ceftazidime	3	8.8	3	8.8	28	82.4
Cefepime	0	0.0	0	0.0	34	100.0
Imipenem	11	32.4	7	20.6	16	47.1
Meropenem	34	100.0	0	0.0	0	0.0
Ciprofloxacin	0	0.0	0	0.0	34	100.0
Levofloxacin	0	0.0	0	0.0	34	100.0
Amikacin	29	85.3	0	0.0	5	14.7
Gentamicin	29	85.3	0	0.0	5	14.7
Minocycline	0	0.0	2	5.9	32	94.1
Polymyxin B	28	82.4	1	2.9	5	14.7
Trimethoprim/Sulfamethoxazole	0	0.0	0	0.0	34	100.0

### Antibiotic sensitivity of Ralstonia pickettii by the broth microdilution method

All of 48 strains were sensitive to Cefuroxime, Ceftriaxone sodium and Trimethoprim/Sulfamethoxazole, followed by Ampicillin/Sulbactam (97.0%), Cefepime (97.0%), Levofloxacin (97.0%) and Cefuroxime axetil (93.9%). However, all the strains were resistant to Ampicillin, Aztreonam, Cefazolin, Cefotetan, Ceftazidime and Nitrofurantoin. while most of the strains were found to be less responsive to Imipenem (3.0%), Meropenem (3.0%) and Piperacillin (3.2%) (Table 7).

**Table 7:** The antibiotic sensitivity of *Ralstonia pickettii* by broth microdilution method (%)

Antibacterial Drugs	Drug resistant		Intermediary agent		Sensitive	
	Strain numbers	Resistance rate	Strain numbers	Intermediate rate	Strain numbers	Sensitive rate
Ampicillin	32	97.0	1	3.0	0	0.0
Ampicillin/Sulbactam	0	0.0	1	3.0	32	97.0
Piperacillin	29	93.5	1	3.2	1	3.2
Piperacillin/tazobactam	0	0.0	22	88.0	3	12.0
Aztreonam	33	100.0	0	0.0	0	0.0
Cefazolin	33	100.0	0	0.0	0	0.0
Cefuroxime	0	0.0	0	0.0	33	100.0
Cefuroxime axetil	0	0.0	2	6.1	31	93.9
Cefotetan	33	100.0	0	0.0	0	0.0
Ceftriaxone sodium	0	0.0	0	0.0	33	100.0
Ceftazidime	1	3.0	32	97.0	0	0.0
Cefepime	1	3.0	0	0.0	32	97.0
Imipenem	6	18.2	26	78.8	1	3.0
Meropenem	32	97.0	0	0.0	1	3.0
Ciprofloxacin	1	3.0	17	51.5	15	45.5
Levofloxacin	1	3.0	0	0.0	32	97.0
Nitrofurantoin	33	100.0	0	0.0	0	0.0
Amikacin	31	93.9	0	0.0	2	6.1
Gentamicin	31	93.9	0	0.0	2	6.1
Tobramycin	31	93.9	0	0.0	2	6.1
Trimethoprim/Sulfamethoxazole	0	0.0	0	0.0	33	100.0

### Clinical features, treatment and outcome of the infected patients

The average age of the 30 patients infected with *Ralstonia pickettii* was 88 years old, and 43.3% of the patients were above 90 years old. The most common primary diseases were Chronic Obstructive Pulmonary Disease and Coronary disease. The clinical characteristics were listed in Table 8. All the cases suffered from cough and sputum. Nearly 96% of the patients suffered from shortness of breath and pulmonary rales, followed by fever (76.7%) and chest pain (6.7%).

**Table 8:** The clinical manifestations and the positive rates of 30 patients

Variables	Items	Cases	Positive rate (%)
Clinical manifestations Laboratory examinations Chest radiographic sign Underlying disease First-listed diagnoses	Fever Cough sputum Shortness of breath Chest pain Pulmonary rales WBC>10 × 109/L NEUT%>70% CRP>0.8 mg/dl Pulmonary infiltration unilateral Bilateral Pleural effusion unilateral Bilateral HIV Diabetes Chronic renal disease Chronic liver disease Cancer Pneumonia ARDS Lung cancer Cardiac failure	23 30 28 2 29 14 26 25 8 25 7 12 2 25 20 14 3 21 5 3 1	76.7 100 93.3 6.7 96.7 46.7 86.7 83.3 26.7 83.3 23.3 40.0 6.7 83.3 66.7 46.7 10.0 70.0 16.7 10.0 3.3

*Notes* WBC – white blood cell, NEUT% – neutrophil percentage, CRP – C-reactive protein.

ARDS – Acute Respiratory Distress Syndrome.

As for laboratory examination results, only about half of patients had abnormal white blood cell (WBC) count results; however, around four-fifths had a higher neutrophil percentage (NEUT%) and an elevated C-reactive protein (CRP) concentration. The most common chest radiographic sign was bilateral pulmonary infiltration.

The antibiotics used empirically within 14 days before the positivity of *Ralstonia pickttii* culture were extensive. Nearly all the patients were treated with combination antibiotics. 73.3% of the patients used meropenem for the broad antibacterial spectrum, potent antibacterial properties and fewer adverse events, which characteristics were very important for elderly patients with pneumonia of unclear bacteria. 60% of the patients used cephalosporins, followed by quinolones(56.7%), which was thought to have more adverse reactions like QT prolongation. The results were shown in Table 9. After the results of bacteria identification and susceptibility tests returned, treatments were adjusted. Through clinical therapy, 73.3% of the patients obviously improved, while the mortality rate was 13.3% in thirty-days.

**Table 9:** Antibiotics used in patients within 14 days before the positivity of *Ralstonia pickttii* culture

Antibiotics	Cases	Usage rate (%)
Carbapenems 2–4 generation cephalosporins Quinolones Nitromizoles Linezolid β-lactam/β-lactamase inhibitor Glycopeptides Tetracyclines Macrolides Lincomycin	22 18 17 12 10 8 4 2 1 1	73.3 60.0 56.7 40.0 33.3 26.7 13.3 6.7 3.3 3.3

### A two-stage MLST typing system was constructed based on16S rDNA

**Fig. 3:**
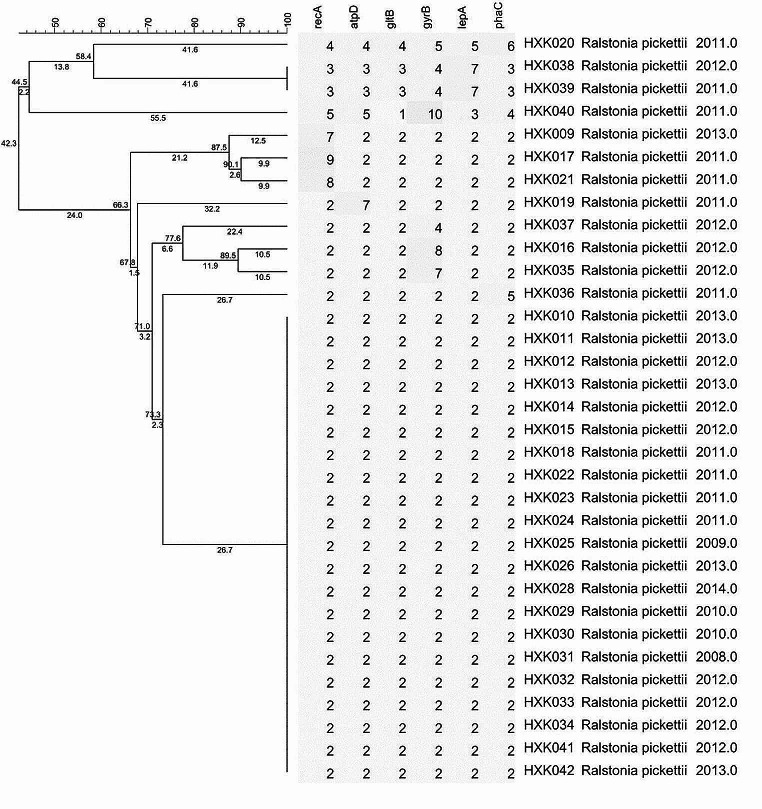
Typing with the species on *Ralstonia pickettii* by MLST

**Fig. 4:**
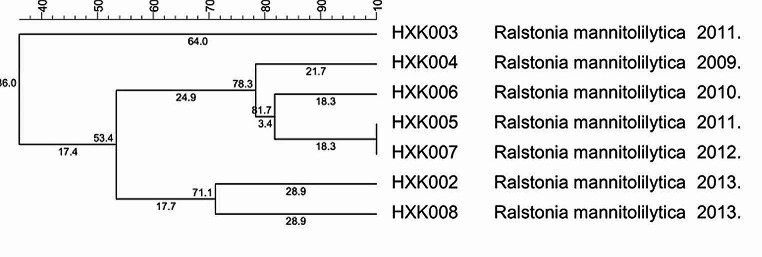
Typing with the species on *R. mannitolilytica* by MLST

We have constructed a two-stage MLST typing system based on16S rDNA. Firstly, the strains were identified by 16 S rDNA, and then a typing of the strains within the species was performed to reach a high-resolution typing effect, thus achieving a quick origin-tracing of the infected strains inside the hospital. The current 16 S rDNA-MLST two-stage typing system had a relatively fine resolution (Figs. 3 and 4).

## Discussion

*Ralstonia* species are non-fermented Gram-negative bacillus which can cause a variety of infections and even outbreaks in hospitals [4, 19]. The *Ralstonia pickettii*, *R. mannitolilytica* and *R. insidiosa* have been reported to be the most common clinically pathogenic pathogens with mis-dentification in clinical laboratory [13]. In our study, only 34 of the 48 clinical strains identified to be *Ralstonia pickettii* with VITEK 2 system were confirmed by the 16 S rDNA method, with an accuracy of only 70.83%. This indicates that the identification results of *Ralstonia pickettii* by conventional methods are not wholly reliable and should be confirmed with molecular assays.

Tom Coenye et al. performed a PCR experiment to identify *Ralstonia pickettii*, *R. mannitolilytica* and *R. insidiosa* using designed primers Rp-F1/Rp-R1, Rm-F1/Rm-R1 and Rp-F1/R38-R1 [13, 16]. It showed that there were corresponding target bands after the amplification of *Ralstonia pickettii* and *R. mannitolilytica* with common primers. Due to the crossing between primers in the identification of *Ralstonia pickettii* and *R. insidiosa*, two target bands showed up in *R. insidiosa*. Our results were complied with that of Tom Coenye’s [15, 16]. Michael P Ryan’s [20] also got the same results. In addition, the 16 S rDNA method was also conducted as a standard method to evaluate the accuracy of the PCR assays. The sensitivity and specificity of the PCR assays about *Ralstonia pickettii*, *R. mannitolilytica* and *R. insidiosa* were 85.3% and 85.7%, 100% and 100%, 71.4% and 90.2%, respectively. In comparison with the previous studies, the sensitivity and specificity are not significantly increase. This is considered to correlate with the types of bacteria selected to evaluate the accuracy of the experiment. There were only 27 strains of *Ralstonia pickettii* and 34 strains of *R. mannitolilytica* out of those 152 strains selected for study by Tom Coenye et al [15]. The other 91 (59.9%) strains had relatively low species correlation with *Ralstonia* which were more easily and correctly to be identified. This study also showed that the 6 strains of non-*Ralstonia* were all identified correctly. Therefore, the PCR assays may be a reliable and simple method to identify *Ralstonia pickettii*, *R. mannitolilytica* and *R. insidiosa*.

Currently, there are fewer studies about the genotyping of *Ralstonia* [21]. Therefore, a standard MLST typing program of *Ralstonia* remains unestablished. Our study designed primers and determined the nucleotide sequence and performed an analysis of genotyping. The results showed that 42 strains of *Ralstonia* contained 18 STs and the most frequent ST was ST9 with 22 strains which are considered as the advantage type and the epidemic strain in our hospital. ST9 strains caused infections each year from 2008 to 2013. UPGMA results indicated that 42 strains of *Ralstonia* were grouped into 2 groups (A and B). Literature has reported identification difficulties of *R. insidiosa* and *Ralstonia pickettii* due to the close similarity [10]. As the cluster analysis shows, the *R. insidiosa* was in ST18, group B had a close genetic relationship with *Ralstonia pickettii*. But in this study, its ST code was 1-1-2-1-1-1 which was obviously different from those of the other strains in this study and consequently can be easily distinguished. However, the ST code of strain HXK003 and HXK042 were both 2-2-2-2-2, the identification result showed that they’re *R. mannitolilytica* and *Ralstonia pickettii* respectively. Our study failed to distinguish them. Due to the inconsistency between the identification result by 16 S rDNA and MLST results, it is considered to construct a two-stage MLST typing system based on 16 S rDNA. What’s more, because the strains of single source are always similar, additional gene locus are needed in the request for higher resolution, which should be further evaluated and screened.

Systematic antibiotic susceptibility data for *Ralstonia Spp* are scarce. Nevertheless, several studies and case reports suggested that most *Ralstonia Spp* are susceptible to commonly used antibiotics [22–24], but the result of the antibiotic susceptibility test using E-tests and broth microdilution method on 53 strains of *Ralstonia pickettii* by Ryan MP showed that the strains had high resistance to gentamicin and aztreonam, different degrees of resistance to ticarcillin/clavulanic acid and had sensitivity to antibiotics such as quinolones, tetracyclines, cephalosporins and sulfamido. Most of the strains were sensitive to Meropenem [22]. However, the results did not quite agree with our study. Almost all *Ralstonia pickettii* isolates were susceptible to cefepime, levofloxacin and Trimethoprim/Sulfamethoxazole and highly resistant to amikacin, gentamicin, aztreonam and meropenem both with the disc diffusion tests and broth microdilution method. However, poor correlation was found with piperacillin, piperacillin/tazobactam, imipenem and ciprofloxacin between MIC and disc diffusion results. Similar variation was found in the results of Ryan MP [22] and a comparable study that was carried out on *Stenotrophomonas maltophilia* [25, 26].

However, there was another interesting finding. As carbapenems, the susceptibilities of strains to imipenem and meropenem were strikingly different both with the disc diffusion tests and broth microdilution method. The isolates showed significantly high resistant to meropenem while much less resistant to imipenem. Especially for the disc diffusion tests, nearly half of the isolates were susceptible to imipenem, but none of the isolates were susceptible to meropenem, which might be attributed to the heavy use of meropenem in our hospital. That might indicate *Ralstonia pickettii* can acquire drug resistance in some way under the antibiotic pressure, which needs further studies.

In our study, we retrospectively analyzed the clinical data of 30 elderly patients infected with *Ralstonia pickettii*. According to our results, the most common features of patients were cough, sputum, shortness of breath, pulmonary rales, high blood NEUT%, CRP concentration and bilateral pulmonary infiltration. These findings were consistent with hospital acquired pneumonia (HAP) in elderly patients and did not have any specific characteristics. Empirical treatment for *Ralstonia pickettii* infection had not yet reached any consensus. For *Ralstonia pickettii* strains that were less responsive to multiple antibiotics in our study, the strategy of combination antibiotics was proven to be useful, but it might also give rise to the appearance of multidrug-resistant bacteria. *Ralstonia pickettii* is an opportunistic pathogen especially in the hospital setting. It is a waterborne microorganism that can survive in any kind of water source and tends to form and maintain biofilms. Most of the patients in this study were elderly and had a long hospital stay. Long-term medical interventions, including the use of broad-spectrum antibiotics, deep venous catheterization, dialysis and mechanical ventilation, increased the risk of infection with Ralstonia pickettii, especially in patients infected with multi-drug resistant bacteria. These factors may lead to infections and the difficulties of removing of Ralstonia pickettii. However, our results have some limitations. The incidence and drug-resistant of *Ralstonia pickettii* could vary among different countries, regions, or even hospitals, giving rise to significant bias. Moreover, the clinical data was collected retrospectively so that some details on patients’ symptoms could be lost. Therefore, further researches are still needed to discover more detailed features of *Ralstonia pickettii* infections.

The specific PCR experiment in this study was reliable and simple, therefore, it can be used as a method to identify the clinically pathogenic bacteria of *Ralstonia* and provide references for clinical diagnosis. This study tried to investigate the MLST typing on *Ralstonia* and preliminarily establish *Ralstonia* ‘s MLST system to provide references for its genotyping to facilitate *Ralstonia* ‘s origin-tracing of its infections, especially outbreaks inside the hospital, as well as the study on its population structure. There was no specific clinical manifestations for *Ralstonia pickettii* pneumonia, the antibiotics should be chosen in reason according to the result of the drug susceptibility test due to *Ralstonia pickettii*’s resistance to various antibiotics.

## Data Availability

The data underlying this article will be shared on reasonable request to the corresponding author.
